# Multimodal Functional Assessment of Asymmetries in Youth Soccer Players: Study Protocol

**DOI:** 10.3390/life16060876

**Published:** 2026-05-24

**Authors:** Ada-Maria Codreanu, Dan-Andrei Korodi, Nicoleta-Alexandra Lupu, Anca-Valentina Onciulenco, Andreea-Ancuta Vataman, Adina-Octavia Duse, Marius-Zoltan Rezumes, Elena-Constanta Amaricai, Liliana Catan, Alexandru Caraba, Roxana-Ramona Onofrei, Claudia Borza

**Affiliations:** 1 Doctoral School, “Victor Babeș” University of Medicine and Pharmacy, 300041 Timișoara, Romania; ada.codreanu@umft.ro (A.-M.C.); nicoleta.onica@umft.ro (N.-A.L.); anca.onciulenco@umft.ro (A.-V.O.); andreea.vataman@umft.ro (A.-A.V.); marius.rezumes@umft.ro (M.-Z.R.); 2 Department of Functional Sciences—Pathophysiology, Center for Translational Research and Systems Medicine, “Victor Babeş” University of Medicine and Pharmacy, Eftimie Murgu Sq. No. 2, 300041 Timişoara, Romania; borza.claudia@umft.ro; 3 Department of Medicine, Faculty of Medicine, “Vasile Goldiș” Western University of Arad, L. Rebreanu St. 86, 310048 Arad, Romania; korodi.andrei@uvvg.ro; 4 Department of Rehabilitation, Physical Medicine and Rheumatology, “Victor Babeș” University of Medicine and Pharmacy Timișoara, Eftimie Murgu Sq. No. 2, 300041 Timișoara, Romania; duse.adina@umft.ro; 5 Department of Rehabilitation, Physical Medicine and Rheumatology, Research Center for Assessment of Human Motion, Functionality and Disability, “Victor Babeș” University of Medicine and Pharmacy Timișoara, Eftimie Murgu Sq. No. 2, 300041 Timișoara, Romania; amaricai.elena@umft.ro (E.-C.A.); catan.liliana@umft.ro (L.C.); 6 Department of Internal Medicine, Diabetes and Systemic Rheumatology, “Victor Babeş” University of Medicine and Pharmacy, Eftimie Murgu Sq. No. 2, 300041 Timişoara, Romania; caraba.alexandru@umft.ro

**Keywords:** soccer, youth athletes, postural balance, muscle strength, ankle dorsiflexion, limb dominance, surface electromyography, functional assessment, force platform, biomechanics

## Abstract

Background: Youth soccer players are exposed to repeated unilateral loading during a period of rapid growth and neuromuscular maturation. These demands may contribute to postural deviations and inter-limb functional asymmetries that can influence movement control and mechanical efficiency. This study protocol aims to establish a standardized multimodal framework for assessing postural alignment, postural control, lower limb mechanical output, ankle dorsiflexion strength, support-limb neuromuscular activation, and contextual training and recovery variables in licensed youth soccer players aged 13 to 17 years. Methods: This prospective observational study will include 75 male youth soccer players recruited from S.C. Fotbal Club Ripensia Timișoara S.A. The primary outcome is the inter-limb asymmetry index derived from unilateral countermovement jump performance. Secondary outcomes include postural alignment, balance, bilateral jump performance, ankle dorsiflexion strength, and support limb electromyographic activity during the instep kick. Participants will complete a clinical evaluation questionnaire, including demographic, training, and recovery variables. Assessments will be conducted using the GaitON system, Kinvent K-Delta force platforms, K-Myo surface electromyography, and K-Pull dynamometry, before and after a regular training session. Biological maturation will be estimated using the Mirwald maturity offset method. Expected Results: The protocol will allow characterization of inter-limb asymmetries across postural, balance, jump, and electromyographic parameters. Conclusions: This protocol aims to provide a practical and standardized model for functional screening in youth soccer players.

## 1. Introduction

Soccer exposes children and adolescents to repetitive unilateral mechanical demands during a critical period of growth and neuromuscular maturation [1]. Young players frequently perform actions such as kicking, cutting, and single-leg landings, which load the musculoskeletal system asymmetrically and favor the development of limb dominance [2]. During adolescence, rapid changes in body size and motor control increase susceptibility to postural deviations and functional imbalances [3]. Biological maturation influences neuromuscular performance, postural control, and inter-limb asymmetries during adolescence [4]. Players of the same chronological age may differ in maturation status, especially around peak height velocity (PHV), which affects strength, coordination, and movement mechanics [4]. Maturity offset, estimated from anthropometric variables, allows classification into pre, circa, or post PHV. The Mirwald maturity offset equation provides a practical and non-invasive method for this purpose [5]. Maturity offset will also be included in the analysis as a stratification variable to account for differences in biological development between players.

Postural deviations and functional asymmetries are frequently reported in young athletes, particularly in sports characterized by repetitive asymmetric tasks [2]. These deviations include pelvic tilt, spinal deviations, shoulder asymmetry, and altered lower limb alignment, which influence global movement mechanics and load distribution [3]. In youth soccer players, limb dominance represents a key determinant of body asymmetries, especially at the level of the lower extremities [2,3].

Inter-limb asymmetries in muscle strength and power are widely described in athletic populations and are often discussed in relation to performance and movement efficiency. Values exceeding 10 to 15 percent are commonly reported in the literature, although their interpretation depends on the task and population studied [1]. In youth soccer, higher asymmetry levels are often observed at younger ages and tend to decrease with maturation and structured training exposure [4,6].

Countermovement jump (CMJ) testing provides objective information on lower limb power and neuromuscular coordination. Single-leg CMJ assessments are considered more sensitive than bilateral tests for identifying functional asymmetries and sport-specific loading strategies [7]. Associations between asymmetry indices derived from single-leg CMJ and strength measures further support their use in functional screening protocols [7,8].

Balance and postural control are essential components of soccer performance and are linked to injury risk. Younger athletes often display reduced postural stability and greater inter-limb differences compared to older adolescents, reflecting ongoing sensorimotor development [9,10]. Portable force platforms provide valid and reliable measurements of center of pressure parameters and allow field-based assessment of balance and mechanical output [11].

The instep kick represents a highly asymmetric movement that requires a stable support limb to ensure effective force transfer and movement accuracy [12]. The tibialis anterior (TA) contributes to ankle stabilization, control of stiffness during single-leg stance, and foot positioning. In the present protocol, we selected TA for anatomical and stability reasons as a representative muscle for surface EMG (sEMG) assessment using the K-Myo system. We acknowledge this as a limitation of the study. However, other muscles, including the gastrocnemius medialis, peroneus longus, and gluteus medius, also play important roles in supporting limb stability [13].

Isometric ankle dorsiflexion strength testing provides information on dorsiflexor force capacity, with relevance for balance and lower limb control in soccer-specific actions [14,15,16].

Static postural assessment adds information on segmental alignment and frontal plane asymmetries. The GaitON motion analysis system has demonstrated good reliability for posture assessment in healthy populations [17].

Studies have evaluated isolated components of asymmetry, such as postural alignment, strength, jump performance, or balance, without examining their interaction within an integrated framework [2,7,8,9]. This limits the understanding of how structural alignment, neuromuscular control, and functional performance contribute to the development of asymmetry in youth soccer players. The selected screening assessments provide both specific and overall perspectives on lower limb function, collectively contributing to a comprehensive biomechanical and functional profile of young soccer players.

The aim of this study protocol is to establish a standardized multimodal screening protocol for assessing postural and neuromuscular asymmetries in licensed youth soccer players aged 13 to 17 years. The protocol integrates static postural assessment using the GaitON system, balance and jump analysis using Kinvent force platforms, ankle dorsiflexion strength testing using Kinvent K-Pull, electromyographic evaluation of the support limb during the instep kick, and contextual data collected through a clinical evaluation questionnaire.

It is hypothesized that inter-limb asymmetry indices exceeding 10 to 15 percent in single-leg CMJ power will be observed, with higher values expected in players aged 13 to 14 years compared to those aged 15 to 17 years. Higher asymmetry values are expected to be associated with lower balance performance and lower ankle dorsiflexion strength, consistent with previously reported thresholds and associations in the athletic population [1].

## 2. Materials and Methods

This protocol study aims to evaluate postural and functional asymmetries in licensed youth soccer players, with the objective of establishing a standardized functional screening model applicable in youth soccer. The protocol focuses on biomechanical and neuromuscular characteristics related to limb dominance, postural control, lower limb strength, jump performance, and support limb stability during the kicking action. In addition to instrumental measurements, the protocol includes the collection of contextual variables related to training exposure and recent recovery status.

The study is designed as a prospective, observational, non-interventional protocol. Data collection will take place within an affiliated football club in Romania, between March 2026 and June 2026. All assessments are non-invasive and reflect standard functional screening procedures commonly used in sports medicine and performance environments.

At the beginning of the testing session, each participant will complete a clinical evaluation questionnaire (Appendix A Table A1). This form records demographic data, anthropometric characteristics, dominant leg, football-related variables, recent match exposure, sleep duration, perceived general condition, training intensity, post-training pain, and recent lower limb injury history. These data will be used for eligibility screening, sample description, and exploratory analysis of contextual factors that may influence functional performance and asymmetry outcomes.

All measurements will be performed by the same trained investigators to reduce inter-examiner variability. Assessments will be conducted before and after a training session to allow the evaluation of changes in neuromuscular and postural parameters. The training session will be characterized by recording the duration and structure, respectively, as well as the warm-up, technical drills, tactical exercises, and small-sided games, to describe the training stimulus across participants. A standard training session for licensed youth soccer players has a structured format adapted to age. The session includes a 10 to 15 min warm-up with running, mobility, and activation, a 15 to 25 min technical phase with ball drills, a 20 to 30 min tactical phase with small-sided games, and a 5 to 10 min cool-down with running and stretching. Weekly training frequency and recent match exposure will be recorded through the clinical evaluation questionnaire. Training sessions follow a consistent structure within each age group, which reduces variability in training stimulus across participants. Internal load measures such as session RPE were not available in the current setting.

This protocol is designed to ensure methodological transparency and reproducibility and to provide a framework for subsequent analyses of postural control, inter-limb asymmetries, and neuromuscular activation patterns in youth soccer players. Results derived from this protocol will be reported in a subsequent outcomes study.

### 2.1. Sample Size Calculation

Sample size estimation was performed using G Power software version 3.1.9.7 (Heinrich Heine University Düsseldorf, Düsseldorf, Germany). The calculation was based on paired comparisons between dominant and non-dominant limbs for the primary outcome, defined as the inter-limb asymmetry index derived from unilateral CMJ performance.

A moderate effect size of 0.50 was selected based on previous studies investigating inter-limb asymmetries in youth soccer players and athletic populations using single-leg CMJ and strength-related outcomes [7,8]. This estimate was supported by a recent single-leg CMJ asymmetry study that used an effect size of 0.5 with statistical power of 0.80 and an alpha level of 0.05 for sample size estimation [7]. This value was considered appropriate as a conservative estimate for paired limb comparisons in the adolescent population with expected variability in neuromuscular performance. The type I error probability was set at α = 0.05, and statistical power was set at 0.80. For a two-tailed paired samples comparison, the minimum required sample size was 64 participants.

To increase statistical robustness and to account for potential variability in measurements or incomplete datasets, a total of 75 participants will be recruited. This sample size is expected to provide sufficient power for detecting meaningful inter-limb differences at the group level.

Analyses according to age category and playing position will be considered exploratory and will not be separately powered.

### 2.2. Recruitment and Informed Consent

Participants will be recruited from licensed youth soccer teams, specifically from S.C. Fotbal Club Ripensia Timișoara S.A. Eligible athletes and their legal guardians will receive detailed verbal and written information regarding the purpose, procedures, duration, and non-invasive nature of the study.

Written informed consent will be obtained from the parents or legal guardians of all participants, while athletes will provide written assent before participation. Participation is voluntary, and each participant can withdraw at any time without consequences for training or team involvement. At recruitment, each participant will complete a clinical evaluation questionnaire.

The study will be conducted in accordance with the Declaration of Helsinki and has been approved by the Research Ethics Committee of the “Victor Babeș” University of Medicine and Pharmacy Timișoara, Romania (approval no. 26/09.03.2026). The protocol was not prospectively registered due to its observational and non-interventional design.

#### 2.2.1. Inclusion Criteria

Participants must meet the following criteria: male youth soccer players aged between 13 and 17 years, registered in a youth team of S.C. Fotbal Club Ripensia Timișoara S.A., minimum training experience of two years, regular participation in organized team training at least 3 times per week, active participation in official competitions during the current season, ability to perform all functional and biomechanical tests required by the study protocol.

#### 2.2.2. Exclusion Criteria

Athletes will be excluded if they present mixed limb dominance, a lower limb injury within the previous three months, a history of lower limb surgery, neurological disorders, vestibular disorders, musculoskeletal conditions that limit full participation in training or testing, or refusal to provide informed consent.

#### 2.2.3. Discontinuation Criteria

Participants will be excluded from further participation if an injury occurs on the testing day or during the regular training session, as assessments are conducted before and after the same training session. Withdrawal will also occur in cases of non-compliance or voluntary withdrawal of consent.

### 2.3. Study Procedure

Assessments will be conducted during a single testing session and repeated before and after a training session to evaluate changes in neuromuscular and proprioceptive parameters. The fixed assessment sequence will be maintained for all participants to ensure protocol consistency; therefore, potential order effect cannot be fully disentangled from fatigue-related effects in the pre/post training comparison.

Biological maturation will be estimated using the Mirwald maturity offset equation based on chronological age, standing height, sitting height, and body mass [5]. Participants will be classified as pre, circa, or post peak height velocity, and a maturation status will be used as a stratification variable in subsequent analyses, as maturation has been shown to influence neuromuscular performance and asymmetry outcomes in youth soccer players [4].

The assessment sequence will be as follows: completion of the clinical evaluation questionnaire, static postural assessment, balance assessment, bilateral CMJ, single-leg CMJ, ankle dorsiflexion strength assessment, and electromyographic assessment during the instep kick. The same sequence will be repeated after the training session, with post-training pain and perceived training intensity updated on the clinical evaluation questionnaire. To reduce potential learning effects, participants will perform one familiarization trial followed by three recorded trials, with the best performance retained for analysis [18,19,20].

Primary outcome measures will include inter-limb asymmetry indices derived from single-leg countermovement jump and balance parameters [7,8,18]. Secondary outcomes will include postural alignment variables, tibialis anterior electromyographic parameters, and ankle dorsiflexion strength [13,14].

All measurements will be performed by the same trained investigators.

### 2.4. Measurements

#### 2.4.1. Static Postural Assessment

Postural alignment will be evaluated using the GaitON Posture Analysis System (version 1.1.0, Auptimo Technologies LLP, Dlhi, India), a digital tool designed for static postural assessment based on predefined anatomical landmarks. The system analyzes posture from anterior and posterior views and quantifies segmental deviations and left-right asymmetries in the frontal plane [17]. The GaitON system provides two-dimensional static analysis and does not allow quantification of rotational asymmetries in the transverse plane. Therefore, the analysis is restricted to frontal plane alignment variables.

Assessments will be performed with the participant barefoot, standing in a relaxed upright position, arms alongside the body, and gaze directed forward. Image acquisition is standardized, with the camera positioned at a fixed distance and height. For the purposes of the present study, postural evaluation includes selected anatomical landmarks, with a primary focus on pelvic and lower limb alignment.

**Figure 1:**
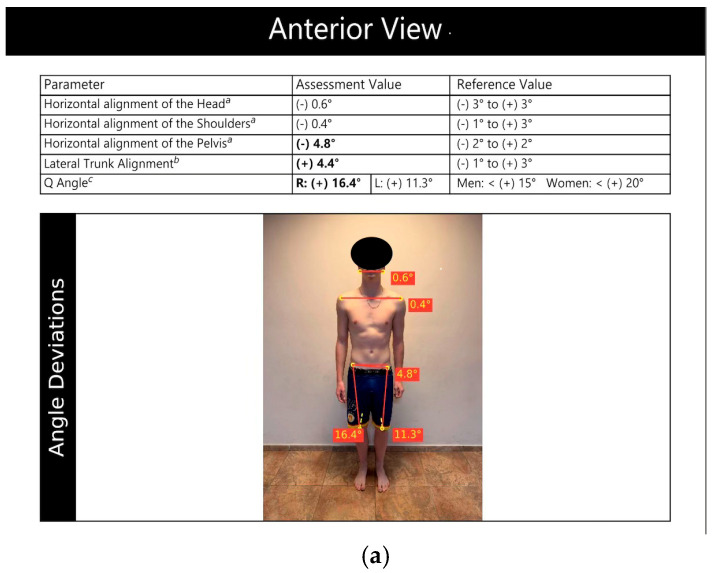

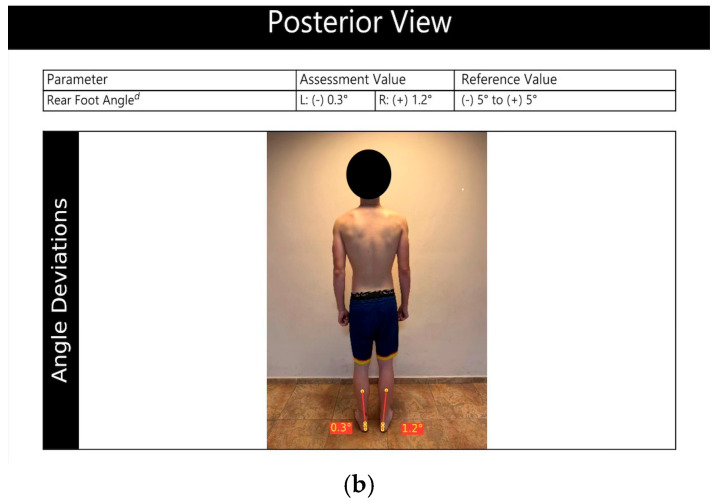
Static postural assessment using the GaitON system (**a**) anterior view: ^a^ horizontal alignment measurements of the head, shoulders, and pelvis; ^b^ lateral trunk alignment; ^c^ Q angle assessment; and ^d^ rear foot angle assessment; (**b**) posterior view: ^d^ rear foot angle assessment (personal archive, published with written parental consent and participant assent).

In the anterior view, anatomical landmarks are placed bilaterally at the level of the earlobe, the anterior superior iliac spine, the midpoint of the patella, and the tibial tuberosity. These anatomical landmarks enable the assessment of pelvic alignment and lower limb axis (Figure 1a). In the posterior view, markers are positioned at the base of the calcaneus, at the insertion of the Achilles tendon, along the central line of the Achilles tendon, and at the mid-calf region. These landmarks facilitate the evaluation of lower limb alignment and foot orientation (Figure 1b). For statistical analysis, the main postural variables retained will include pelvic angle, Q angle, and rearfoot angle as indicators of alignment at the level of the hip, knee, and ankle. Additional variables generated by the software, such as head and shoulder alignment, will be reported descriptively.

Captured images are imported into the GaitON software, where semi-automated analysis is conducted based on the marked reference points [17]. To ensure measurement reliability in this population, a pilot phase will be conducted on a subset of 10 participants. Measurements will be repeated under the same conditions within the same session, with the repositioning of anatomical markers between trials. Intra-rater reliability will be assessed using intra-class correlation coefficients, standard error of measurement, and minimal detectable change [21,22,23,24]. All measurements are performed by the same examiner to ensure consistency.

#### 2.4.2. Balance Assessment

**Figure 2:**
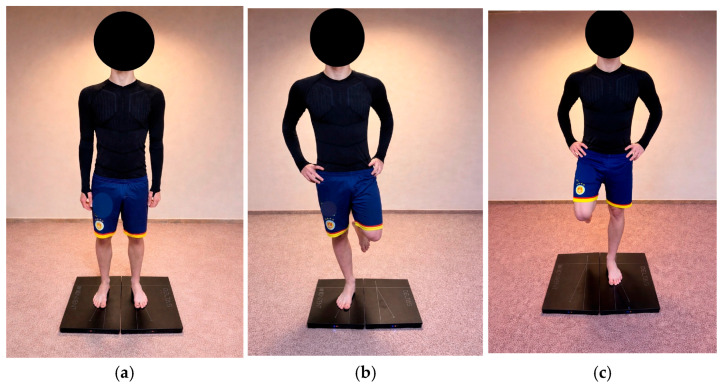
Evaluation of balance using Kinvent K-Delta (**a**) bipedal stance, (**b**) single-leg stance-right leg, and (**c**) single-leg stance-left leg (personal archive, published with written parental consent and participant assent).

Bilateral and unilateral balance will be assessed using Kinvent K-Delta force platforms (Kinvent Biomecanique, Montpellier, France), which provide objective measures of postural control through force and center of pressure variables during static tasks [18]. Assessments are performed with participants barefoot in standardized stance positions. For bilateral balance evaluation, participants stand with both feet symmetrically on the platforms, arms relaxed at the sides, and gaze forward. Static trials last 30 s under eyes open conditions, during which center of pressure, path length, and velocity are recorded (Figure 2a–c). Primary variables retained for analysis include mean center of pressure velocity (CoP) expressed in mm per second, total path length in mm, and ellipse area in mm^2^. Secondary variables include mediolateral and anteroposterior displacement, time to stabilization, and weight distribution between limbs.

Balance assessment will be performed under eyes-open conditions only. An eyes-closed condition was not included in order to maintain consistency across participants and minimize testing duration in a field-based setting. The selected protocol prioritizes feasibility, test reliability, and comparability between limbs, while still allowing the detection of inter-limb asymmetries through force platform measures.

Unilateral balance is assessed separately for dominant and non-dominant limbs. Participants maintain a single-leg stance on the force platform, with the contralateral limb flexed and minimal upper body movement. Center of pressure variables and time to stabilization are recorded to quantify limb-specific postural stability and asymmetry. All trials follow a consistent protocol with standardized durations and rest intervals to minimize fatigue, and data are processed using the Kinvent application version 2.24.0 (Kinvent Biomecanique, Montpellier, France). The K-Delta force platforms have demonstrated validity and reliability for balance assessment, showing comparable measurements with established laboratory force platforms and consistent center of pressure parameters in athletic populations [18]. Participants will perform one familiarization trial followed by three recorded trials, with the best performance retained for analysis.

#### 2.4.3. Strength and Jump Performance

**Figure 3:**
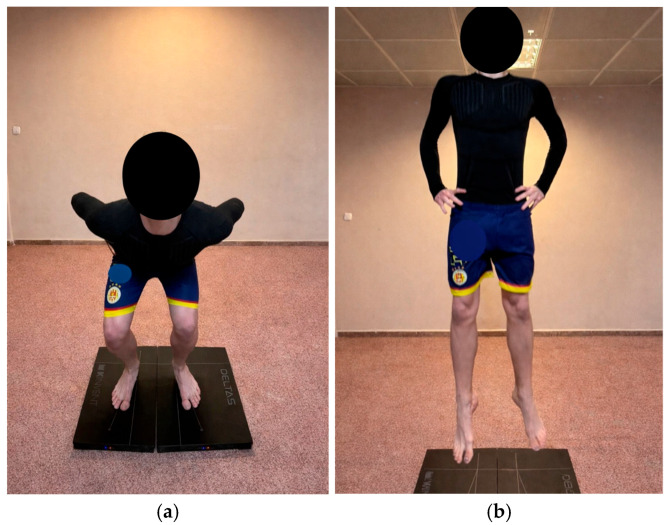
Bilateral countermovement jump (**a**) starting position with hands on hips, (**b**) propulsion phase with extension of hips, knees, and ankles (personal archive, published with written parental consent and participant assent).

**Figure 4:**
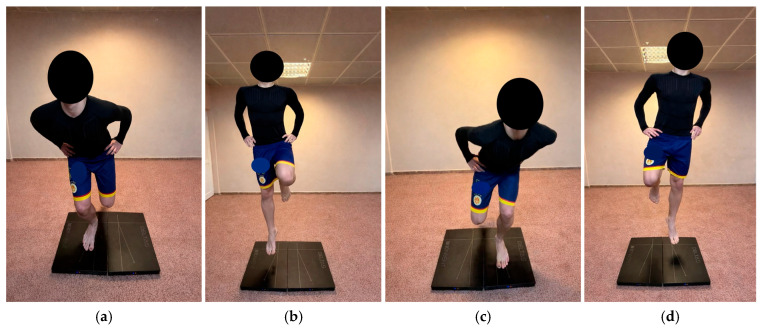
Single-leg countermovement jump (**a**) starting position on the right limb, (**b**) propulsion phase on the right limb, (**c**) starting position on the left limb, and (**d**) propulsion phase (personal archive, published with written parental consent and participant assent).

Lower limb mechanical output and jump performance will be evaluated using bilateral (Figure 3a,b) and single-leg countermovement jump (Figure 4a–d) tests performed on the Kinvent K-Delta force platforms. The system records vertical ground reaction force and provides real-time outputs for peak force, impulse, power, and jump height [18]. For the bilateral countermovement jump, participants stand with their feet shoulder-width apart and their hands on their hips. They perform a rapid downward movement through hip, knee, and ankle flexion, followed by an explosive upward extension to achieve maximal jump height.

For the single-leg countermovement jump, participants stand on one leg with the opposite leg flexed and hands on their hips. They perform a controlled downward phase followed by a rapid extension to jump vertically and land on the same leg. The test is performed separately for each limb. Participants will perform one familiarization trial followed by three recorded trials for each jump condition. A rest interval of 30 s will be provided between trials, and 2 min between testing conditions, in line with commonly used protocols for countermovement jump assessment [20]. The best performance will be retained for analysis.

Primary variables retained for analysis will include jump height, peak force, and peak power. Impulse will be included as a secondary variable to further characterize force production during the concentric phase.

The Kinvent application calculates asymmetry indices based on inter-limb differences in these variables. Asymmetry indices will be calculated using the percentage difference formula, defined as: asymmetry (%) = (|dominant − non dominant|/max value) × 100. This method is widely used in sports science due to its simplicity and suitability for paired limb comparisons, particularly in youth and athletic populations [1,20]. The directionality of the asymmetry will be reported descriptively. This approach allows the objective and sensitive detection of mechanical asymmetries [7].

#### 2.4.4. Kicking Action and Electromyographic Assessment

**Figure 5:**
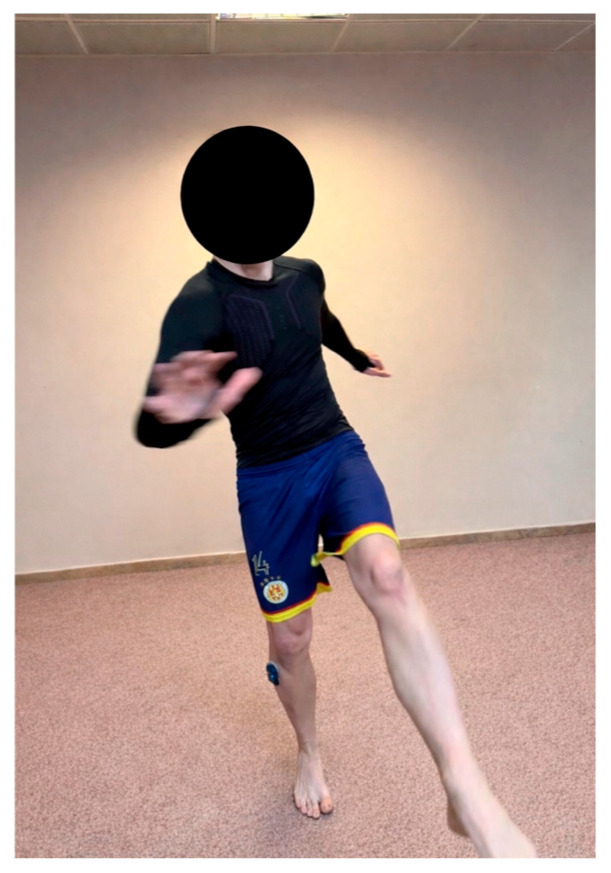
Electromyographic assessment during the instep kick. Surface EMG sensor placed on the tibialis anterior of the support limb during kick (personal archive, published with written parental consent and participant assent).

The stability of the support limb during the instep kick will be evaluated using K-Myo surface electromyography (sEMG), (Kinvent Biomecanique, Montpellier, France) (Figure 5). Signals will be recorded exclusively from the TA muscle of the support limb, selected for its role in ankle stabilization, foot positioning, and regulation of ankle stiffness during single-leg stance and dynamic loading tasks [13]. The TA was also selected because it allows reliable surface EMG acquisition in field conditions [19].

**Figure 6:**
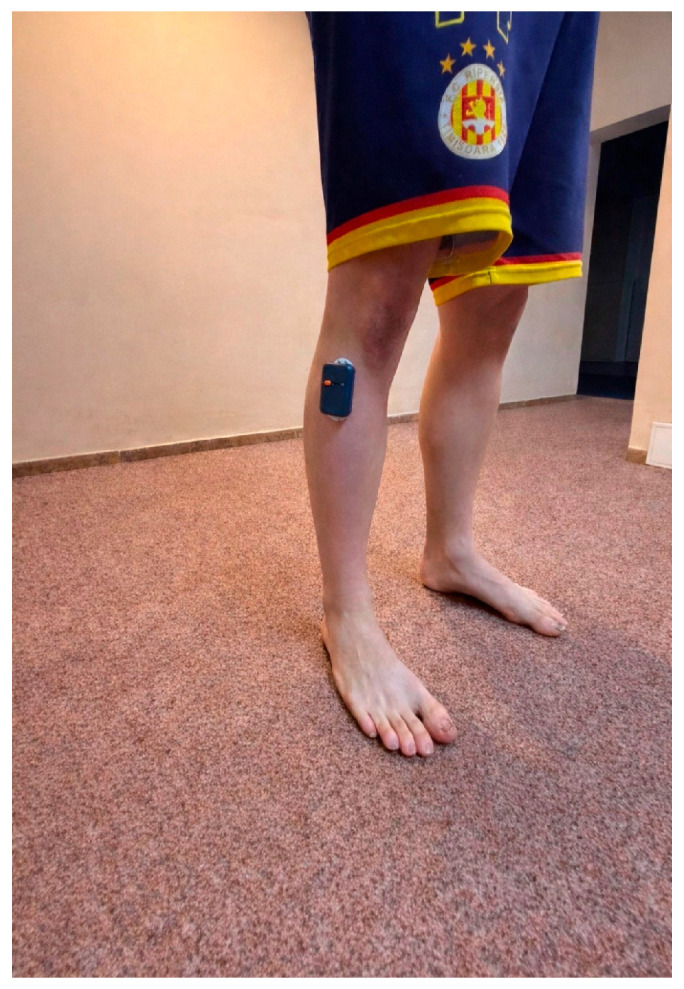
Sensor placement for sEMG. Electrodes positioned over the tibialis anterior muscle according to SENIAM guidelines (personal archive, published with written parental consent and participant assent).

sEMG sensors will be positioned over the muscle belly of the TA muscle (Figure 6) following SENIAM recommendations, after skin preparation consisting of shaving, light abrasion, and alcohol cleaning to reduce impedance [19]. The wireless K-Myo sensors will be secured to the skin using pre-gelled adhesive electrodes and elastic fixation and will transmit data in real time to a tablet via the Kinvent application.

Signals will be recorded using a single differential channel at 1000 Hz, bandwidth 10 to 1061 Hz, with automatic mains interference filtering [25].

Signal normalization will be performed relative to maximal voluntary isometric contraction obtained during ankle dorsiflexion testing using the Kinvent K-Pull system, in accordance with established recommendations for EMG normalization [19,26]. EMG signals will be recorded synchronously during the K-Pull dorsiflexion trials to obtain the MVIC reference value. Primary variables retained for analysis will include normalized root mean square amplitude and peak activation, expressed as a percentage of maximal voluntary contraction. Secondary variables will include activation onset (determined by visual inspection) and activation duration, expressed in milliseconds during the support phase of the kick.

Participants will perform an instep kick using the dominant limb, while electromyographic activity is recorded from the support limb. A standard size five soccer ball will be used, inflated within the recommended pressure range of 0.6 to 1.1 bar according to international guidelines [27]. The ball will be positioned at a fixed distance of 7 m from the target area measuring 1 × 1 m. Participants will perform a short run-up of two to three steps before ball contact.

Kicks will be executed using the full instep. One familiarization trial followed by three recorded trials will be completed under consistent field conditions. The coefficient of variation across the three trials will be calculated for each participant. If the coefficient of variation exceeds 20%, the set of trials will be repeated to ensure measurement consistency. Trials will be considered valid if correct ball contact is achieved and balance is maintained during and after the kick. Trials with evident technical errors, such as missed contact with the ball or loss of balance, will be discarded and repeated. Kick execution will be visually monitored by the evaluators to ensure consistency across trials. The mean of three valid trials will be retained for analysis.

These data will allow the assessment of neuromuscular strategies associated with support limb control during soccer-specific kicking actions [12].

#### 2.4.5. Ankle Dorsiflexion Strength Assessment Using Kinvent K-Pull

**Figure 7:**
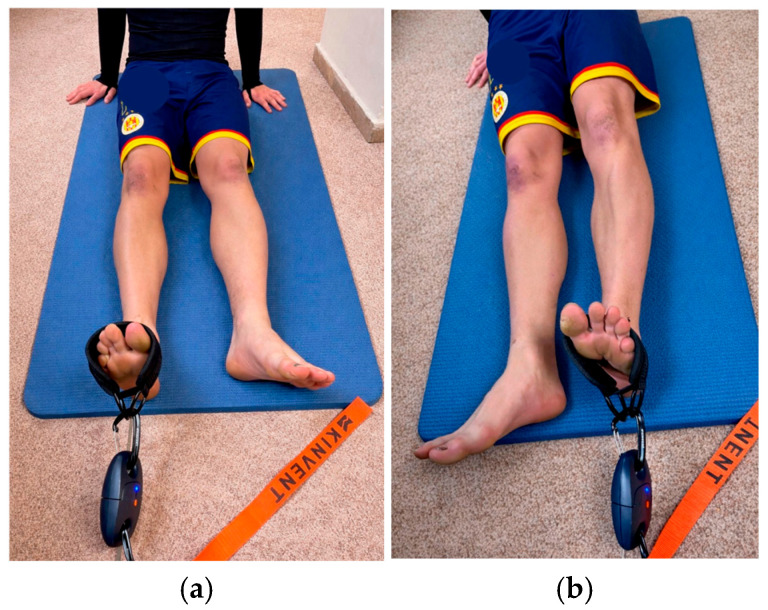
Isometric ankle dorsiflexion assessment (**a**) right limb (**b**) left limb (personal archive, published with written parental consent and participant assent).

Isometric ankle dorsiflexion strength will be assessed using the Kinvent K-Pull system (Kinvent Biomecanique, Montpellier, France), an inline dynamometry solution designed for field-based quantification of force output during strap-fixed isometric tasks. The test is performed separately for each lower limb, in a standardized supine position, to target dorsiflexor force generation with emphasis on TA activation (Figure 7a,b) [15,16].

Participants will lie supine with the hips in neutral rotation and the tested limb extended. The ankle will be positioned at 90°, corresponding to neutral alignment between the foot and the tibia, and this position will be verified using a handheld goniometer prior to each trial. A non-elastic strap will be secured around the forefoot and connected in line with the Kinvent K-Pull device, with the opposite end fixed to a stable anchoring point. Strap alignment will be adjusted to match the direction of ankle dorsiflexion.

After a familiarization contraction, participants will perform maximal voluntary isometric dorsiflexion by pulling the forefoot toward the tibia against the fixed strap, while maintaining heel contact with the support surface. Heel contact will be visually verified by the examiner during each trial. Trials in which heel-off is observed will be discarded and repeated. Each limb will be tested independently, and maximal effort will be sustained for 3 to 5 s, with a rest interval of 30 s between trials.

Force output will be recorded in real time using the Kinvent application. Primary variables retained for analysis will include peak force expressed in Newtons. Secondary variables will include the rate of force development at 100 ms and 200 ms, which are commonly used time intervals for assessing early force production in neuromuscular performance [28]. Three maximal trials will be performed for each limb, and the best performance will be retained for analysis. This setup follows validated dorsiflexion dynamometry protocols, where strap fixation and standardized positioning improve measurement stability and repeatability [15,16]. Between-session reliability has been reported as good to excellent under standardized conditions [28]. Force asymmetry indices between dominant and non-dominant limbs will be calculated and interpreted alongside balance, jump, postural, and electromyographic outcomes.

### 2.5. Statistical Analysis

Statistical analysis will be performed using MedCalc Statistical Software (version 23.2.1, MedCalc Software Ltd., Ostend, Belgium 2026). Descriptive statistics will be calculated for all variables. Normality will be assessed using the Shapiro–Wilk test. A linear mixed-effects model will be used to analyze repeated-measures data, with participant included as a random intercept and limb (dominant vs. non-dominant), time (pre vs. post training), and their interaction included as fixed effects. Differences between age groups and playing positions will be analyzed using analysis of variance or non-parametric equivalents. Differences between age groups and playing position will be analyzed using analysis of variance or non-parametric equivalents. Maturation status categories (pre, circa, and post PHV) will be used as stratification variables in exploratory subgroup analyses. Age groups will be predefined using chronological categories of 13–14 years, 15–16 years, and 17 years. This grouping reflects typical age stratification in youth soccer and allows comparison across developmental stages. To control for multiple comparisons, the Holm–Bonferroni method will be applied. The effect size with 95% CI will be determined using the partial η^2^ for ANOVA, Cohen’s d_z_ for paired comparisons, and rank-biserial correlation for any non-parametric contrasts. Families of tests will be defined based on outcome domains, including postural alignment variables, balance parameters, jump performance variables, electromyographic variables, and ankle dorsiflexion strength measures. Corrections will be applied separately within each family of tests. Statistical significance will be set at *p* < 0.05.

A pilot phase of the study will analyze the intra-rater reliability for all the outcomes. Intra-class correlation coefficient (ICC) with 95% confidence interval will be used. The ICC (3,1), a two-way mixed-effects model with absolute agreement, will be calculated to assess the intra-rater reliability [21,22]. Values less than 0.75 will be considered moderate, between 0.75 and 0.9 will be considered good, and greater than 0.90 will be considered as excellent [22]. Standard error of measurement (SEM) will be calculated according to the formula: $SEM=SDpooled\times \sqrt{1-ICC}$ [23]. The smaller the SEM values are, the more reliable the measurements are [23,24]. The smallest detectable change at a 95% confidence interval (SDC_95_) will also be calculated to assess the magnitude of real change between measurements necessary to exceed error, based on the formula: $SDC95=1.96\times \sqrt{2\;}\times SEM$ [24,29].

Primary outcomes will be used for the main paired comparisons between dominant and non-dominant limbs. Secondary outcomes will include postural alignment variables, specifically pelvic angle, Q angle, and rearfoot angle, together with TA electromyographic parameters and ankle dorsiflexion strength. These variables will be analyzed descriptively and exploratorily according to age group and playing position. Analyses will consider both limb-related and time-related comparisons to explore changes following the regular training session.

Variables obtained from the clinical evaluation questionnaire, including age, playing position, years of practice, match exposure in the previous 7 days, sleep duration, perceived general condition, post-training pain, and perceived training intensity, will be reported descriptively and may be explored as contextual factors in secondary analyses.

## 3. Expected Results

The protocol is designed to characterize the prevalence and magnitude of postural and functional asymmetries in licensed youth soccer players aged 13 to 17 years. Differences between dominant and non-dominant limbs will be examined across balance, strength, jump performance, ankle dorsiflexion strength, and support limb stability during the kicking action [1,7,8].

Age-related differences in asymmetry will be explored across groups, in the context of ongoing neuromuscular maturation [4]. Differences between playing positions will be explored descriptively, and these comparisons are not powered for position-specific inference.

Electromyographic analysis is intended to examine activation patterns of the TA during the support phase of the instep kick, in relation to ankle stability and postural control [13]. The study will explore whether variations in activation timing and amplitude are associated with inter-individual differences in support limb stability.

Isometric dorsiflexion dynamometry using Kinvent K-Pull is designed to detect limb-specific differences in dorsiflexor force capacity that may relate to unilateral balance performance and force platform-derived measures of postural control. Evidence in elite youth soccer suggests that dorsiflexion strength may be associated with balance performance [14]. In the present study, the protocol will explore whether similar associations are observed.

The integration of postural analysis, force platform testing, ankle dorsiflexion dynamometry, and electromyography is intended to provide a comprehensive functional profile of young soccer players [11,18]. The results may support the identification of functional profiles that may be relevant to training adjustment and individualized monitoring in youth soccer players.

This protocol is designed to offer a standardized and reproducible framework for functional screening in youth soccer, with potential applicability in sports medicine, performance monitoring, and long-term athlete development programs.

## 4. Discussion

The present protocol proposes a multimodal assessment of postural and neuromuscular asymmetries in licensed youth soccer players aged 13 to 17 years. Previous studies have often focused on individual components of asymmetry, such as postural alignment, jump performance, or balance [2,7,8,9]. The present protocol integrates postural assessment, balance testing, jump performance, ankle dorsiflexion strength, and electromyographic analysis within a single field-based framework.

Postural assessment was included to quantify frontal plane alignment at the level of the pelvis, knee, and ankle. Theodorou et al. evaluated 76 youth soccer players and reported significant asymmetries related to limb dominance at the lower limb level [2]. Based on these findings, the present protocol uses digital postural analysis to provide objective and standardized measurements of segmental alignment.

Balance and jump performance assessments were selected to evaluate postural control and mechanical asymmetry in youth soccer players. Previous studies have shown that younger athletes present reduced balance performance and greater asymmetry compared to older groups, reflecting ongoing neuromuscular maturation [9]. Single-leg CMJ has also been reported as sensitive for detecting inter-limb differences in force and power, while asymmetry magnitude may differ between bilateral and unilateral jump tasks [7,20]. Based on these findings, the present protocol integrates force platform-derived balance variables together with bilateral and single-leg CMJ to provide a broader characterization of lower limb asymmetry [18].

Ankle dorsiflexion strength was included to assess limb-specific force capacity. Kayhan et al. evaluated elite youth soccer players and reported associations between dorsiflexion strength and balance performance [14]. Olsen et al. reported good to excellent reliability for dorsiflexion strength and rate of force development measures [28]. Torque control during ankle dorsiflexion may also contribute to dynamic postural control, as Nozu et al. reported associations between eccentric dorsiflexion control and dynamic balance performance [30]. Based on these findings, the present protocol includes peak force and rate of force development as strength variables and will explore associations with balance and jump outcomes.

Electromyographic assessment focuses on the tibialis anterior during the support phase of the instep kick. Mayer et al. reported activation of this muscle during single-leg stabilization tasks [13]. sEMG recording follows standardized placement recommendations described by Hermens et al. [19], which supports consistency of data acquisition. These variables will be interpreted in relation to mechanical and balance outcomes.

The interpretation of results will be based on the integration of these domains. Ličen and Kozinc reviewed 31 studies and reported asymmetry thresholds of 10 to 15% in strength and power measures [1]. In the present protocol, asymmetry indices exceeding this range will be interpreted alongside balance, strength, and electromyographic variables. Plantar loading patterns may provide additional information on asymmetry. Azevedo et al. reported that plantar pressure asymmetries are present in young soccer players and may be associated with stress-related injury risk [31]. These findings may complement the interpretation of strength, balance, and electromyographic outcomes in the present protocol.

Portable assessment tools were selected to ensure applicability in field conditions. Mylonas et al. validated portable force platforms and reported good agreement with laboratory systems [11]. This supports the use of Kinvent platforms for applied assessment in youth soccer environments.

Overall, the present protocol integrates multiple functional domains within the same population. This approach allows a multidimensional characterization of asymmetry and supports the identification of functional profiles relevant for monitoring and training in youth soccer players.

An important feature of the present protocol is the inclusion of contextual variables obtained from the clinical evaluation questionnaire, such as recent match exposure, sleep duration, perceived general condition, post-training pain, and perceived training intensity. These variables may strengthen the interpretation of functional findings by reducing the risk of attributing observed differences exclusively to structural asymmetry, when recent fatigue, recovery status, or competitive exposure may also contribute.

From a practical perspective, the proposed protocol may assist clinicians, strength and conditioning specialists, and coaches in identifying inter-limb deficits at an early stage and implementing individualized corrective strategies within youth training environments. This protocol may help practitioners identify athletes with relevant asymmetry profiles and support targeted interventions focused on balance training, strength correction, or neuromuscular control.

### Study Limitations

This study has several limitations that should be acknowledged. The observational design does not allow causal inferences regarding the relationship between postural asymmetries and injury risk or performance outcomes. The study focuses on a single assessment period, which limits the ability to evaluate long-term adaptations or the progression of asymmetries over time. Measurements are performed within a single session, and day-to-day variability is not assessed. Analyses are limited to associations between variables and do not support predictive modeling. In addition, several contextual variables collected through the clinical evaluation questionnaire, including sleep duration, perceived general condition, pain, and training intensity, are partially self-reported and may therefore be affected by recall bias or subjective interpretation.

The sample includes only male youth soccer players, which restricts the generalizability of the findings to female athletes or participants from other sports. The sample includes only male youth soccer players recruited from a single club (S.C. Fotbal Club Ripensia Timișoara S.A.), which may limit the generalizability of the findings even within youth soccer populations due to club-specific coaching methodology, selection criteria, and training philosophy.

Biological maturation will be estimated using the Mirwald maturity offset equation; however, this method provides an indirect estimate of maturation status and may not fully capture individual variability in biological development. Chronological age grouping may not fully reflect biological maturation status, and residual confounding related to maturation differences may influence the interpretation of age-related comparisons.

Although the protocol includes a multimodal set of functional assessments, these measurements may not fully capture movement variability during real match situations. Field-based assessments may introduce environmental variability compared to controlled laboratory conditions. Fatigue is evaluated only before and after a training session and may not reflect cumulative match-related fatigue.

Electromyographic assessment is limited to selected muscles involved in ankle stabilization and does not provide a complete representation of lower limb or trunk muscle activation patterns. Surface electromyography is limited by potential cross-talk from adjacent muscles.

Repeated testing before and after training may introduce learning effects that influence performance outcomes. The use of a fixed testing sequence for all participants may also introduce order or fatigue-related effects, particularly for assessments performed later in the protocol. Position-specific analyses are limited by the relatively small number of participants per subgroup and will be interpreted cautiously.

Finally, the use of multiple testing procedures increases the complexity of data interpretation and may introduce variability related to learning effects or motivation. Despite these limitations, the standardized protocol provides valuable information on postural and functional asymmetries in youth soccer players and offers a practical framework for functional screening in applied sports settings.

## 5. Conclusions

This study protocol proposes a standardized and integrated approach for the functional assessment of postural and neuromuscular asymmetries in licensed youth soccer players aged 13 to 17 years. By integrating static postural analysis, balance testing, strength and jump assessment, ankle dorsiflexion dynamometry using Kinvent K-Pull, and electromyographic evaluation during the kicking action, the protocol addresses key biomechanical and neuromuscular factors relevant to youth soccer.

The data obtained through this protocol are expected to clarify the magnitude and distribution of inter-limb asymmetries across age groups and playing positions. This information may support the early identification of functional deficits and suboptimal movement strategies in young athletes.

The proposed assessment framework may serve as a practical screening tool for sports medicine professionals, coaches, and strength and conditioning specialists. Its implementation could contribute to individualized training adjustments, targeted prevention strategies, and optimized long-term athlete development in youth soccer.

## Data Availability

The data presented in this study are available on request from the corresponding author (R.R.O.) due to privacy considerations.

## Abbreviations

The following abbreviations are used in this manuscript:

CMJ	Countermovement Jump
EMG	Electromyography
K-Myo	K-Myo Surface Electromyography System
K-Pull	Kinvent K-Pull Inline Dynamometry System
TA	Tibialis Anterior

## Appendix A

This questionnaire was designed to systematically capture demographic data, anthropometric characteristics, soccer-specific experience, training load, and match exposure, recent lower limb injury history, as well as subjective indicators including perceived pain, general condition, sleep duration, and training intensity.

**Table A1:** Clinical evaluation questionnaire.

Name and surname
Date of birth
Age (years)
Body mass (kg)
Standing height (cm)
Sitting height (cm)
Leg length (cm)
Dominant leg (left/right)
Playing position (goalkeeper, defender, midfielder, forward)
Soccer practice (years)
Number of training sessions per week and the duration
Minutes played in official matches in the last 7 days
Lower limb injuries in the last 3 months (date, type of injury, and days without training)
Pain after training 0–10 (0 = no pain 10 = severe pain)
General condition (good/average/poor)
Hours slept the previous night
Training intensity (1—low, 2—moderate, 3—high)
